# Irregularities of the Teeth and Their Treatment

**Published:** 1888-02

**Authors:** 


IRREGULARITIES OF THE TEETH AND THEIR TREATMENT, By Eugene S. Talbott, M. D., D. D. S. Publishers: P. Blakiston, Son & Co., Philadelphia, 1888.

This very comprehensive and valuable publication has been written in such a manner as to embrace a clear and practical understanding of the etiology and treatment of dental irregularities; and its contents include the Anatomy of the Maxillaæ and Teeth, the physiology and description of both temporary and permanent teeth, their occlusion and position the etiology of irregularities, hereditary and acquired irregularities. Causes of irregularities, treatment of all forms, and the nature of the force necessary; and a description of the appliances best adapted for correcting the different forms of irregularities to which the teeth are subject. The style of the work is excellent, and it is embellished with one hundred and fifty-two illustrations, and will be universally recognized as a valuable addition to dental literature. The author is to be congratulated on the able manner in which he has treated a very difficult subject.

